# *Clostridium difficile* Associated Disease in a Neurointensive Care Unit

**DOI:** 10.3389/fneur.2013.00082

**Published:** 2013-07-01

**Authors:** Swagata Tripathy, Priya Nair, Michael Rothburn

**Affiliations:** ^1^Department of Trauma and Emergency Medicine, All India Institute of Medical Sciences, Bhubaneswar, India; ^2^Walton Centre of Neurosciences, Liverpool, UK; ^3^Aintree University Hospital, Liverpool, UK

**Keywords:** *Clostridium difficile*, *Clostridium* infections, neurocritical care, nosocomial infections, toxin A, toxin B, pseudomembranous colitis

## Abstract

**Background:** Critically ill patients are at high risk for acquiring *Clostridium difficile* infection. The aim of this study was to investigate the prevalence, severity, and outcome of neurointensive care unit (NICU) acquired *Clostridium difficile* associated disease (CDAD).

**Materials and Methods:** Intensive care admission and hospital infection control databases from April 2008 to August 2010 were studied and the case notes reviewed retrospectively. Diarrhea was classified as mild, moderate, or severe based on the frequency and volume. Information on demographics, risk factors for CDAD, presentation, and course of the disease was gathered. Admission diagnosis, days of NICU stay, and incidence of complications were noted.

**Results:** In the time period studied, 9 out of 2212 patients (prevalence rate 0.4%) admitted to the intensive care unit (ICU) for a total of 10,825 bed days (incidence rate 8.3 per 10,000 bed days) acquired CDAD. Median age was 55 (IQR 20–72) years. The median NICU stay was 26 (IQR 11–103) days. The median duration between ICU admission and development of CDAD was 11 (IQR 3–93) days. Four patients (44%) had moderate CDAD. Concurrent infections occurred in seven (77%) patients. The most frequently prescribed antimicrobials prior to CDAD were cephalosporins (71%). The apparent risk factors in this group included age>65 year (22%) and antibiotics (67%) among others. One patient developed CDAD colitis. Three patients had a perceived delay in discharge from the ICU (1–8 days) due to their infective status. No mortality was ascribed to CDAD.

**Conclusion:** The prevalence rate (0.4%) and morbidity of CDAD in the unit are low. A larger database is needed to better analyze the associated risk factors in this subgroup of patients. A possible increase in disease burden due to a delay in discharge from the ICU merits further evaluation.

## Introduction

*Clostridium difficile* infection (CDI) and *Clostridium difficile* associated disease (CDAD) is a serious, infectious complication in hospitalized patients. There has been a progressive rise in the incidence, severity, and complications due to CDAD globally over the recent years (Kenneally et al., 2007; Marra et al., 2007).

In the intensive care unit (ICU), CDAD is one of the most important causes of nosocomial infection and has been suggested to be responsible for a 6% incremental increase in the risk of attributable mortality (Kenneally et al., 2007). Although more data is now emerging on CDAD acquired in the general ICU, the information from sub specialty ICU remains scanty (Musa et al., 2010, 2011; Crabtree et al., 2011).

The suggested risk factors for acquiring *C. difficile* infection are prolonged hospital stay, antibiotic usage, advanced age, immunosuppression (corticosteroids), indwelling devices, laxative use, and proton pump inhibitors (Bobo et al., 2011). Some of these may be more prevalent in the neurointensive care unit (NICU) than in other wards. The incidence of, risk factors for, and outcomes of CDAD in this subgroup of patients are poorly studied and remain unclear with only one previous published study describing the prevalence and outcome associated with NICU-acquired CDAD (Musa et al., 2010).

The aim of this study was to assess the epidemiology, clinical course, management, and outcomes of ICU-acquired CDAD among patients admitted our NICU.

## Materials and Methods

The center of interest is a tertiary care referral neurosciences center in the UK with a catchment population of about five million. The NICU was upgraded early in 2010 from a 14- to a 20-bedded setup of which 16 are level 3 beds equipped for patients of multiorgan failure who may require advanced respiratory support. This includes eight isolation rooms where colonized or infected patients are managed. The high dependency area has four level two beds which are occupied by patients requiring basic cardiopulmonary support or admitted for management of a single organ failure. The nurse to patient ratio for ICU beds is 1:1 and for the high dependency area is 1:2. Hand hygiene with soap and water or alcohol hand gel and personal protective equipment such as gloves and plastic aprons are routinely practiced for all patients before and after contact. Regular audits of hand hygiene practice and environmental standards are carried out by the infection control team.

Contact isolation policy in our unit is stringent with immediate isolation of patients who fulfill Bristol stool five or greater into an isolation room without awaiting confirmation of infection by the stool toxin assay. Removal of an infected patient from isolation after completion of the treatment period requires a symptom free interval and is a multidisciplinary decision involving the NICU team, the clinical microbiologist, and the infection control team.

Intensive care unit-acquired CDI was defined by the infection control team as any of the following-watery or unformed stools, according to the Bristol stool chart (Lewis and Heaton, 1997), occurring ≥48 h after ICU admission with a laboratory confirmation of a stool sample positive for *C. difficile* toxin A or B, toxic megacolon, or ileotomy where the specimen is *C. difficile* toxin positive, pseudomembranous colitis revealed by lower gastro-intestinal endoscopy or Computed Tomography, colonic histopathology characteristic of *C. difficile* infection (with or without diarrhea or toxin detection) on a specimen obtained during endoscopy or colectomy or fecal specimens collected post-mortem where the specimen is *C. difficile* toxin positive or tissue specimens collected post-mortem where pseudomembranous colitis is revealed or colonic histopathology is characteristic of *C. difficile* infection.

The microbiological diagnosis was made by enzyme immunoassay (EIA). A positive EIA result was confirmed by a positive glutamate dehydrogenase test (C. diff Quik Chek, Techlab) and also a positive toxin test (Tox A/B Quik Chek, Techlab).

The UK’s Health Protection Agency (HPA) definitions for diarrhea and disease severity were used to classify the disease (Table 1).

**Table 1:** **Grading of severity of *C. difficile* infection**.

Criteria	Mild	Moderate	Severe
Number of liquid stools per day	<4	5–7	>8
Fever	None	37.5–38.5 °C	>38.6 °C
Abdominal symptoms	None	Severe abdominal pain	Signs and symptoms of peritonism
Serum albumin	Normal	Normal	Low<25 g/l
Complications	None	Lower GI hemorrhage with stable blood pressure	Lower GI hemorrhage with unstable blood pressure
			Perforation of colon
			Sepsis due to colitis
			Renal dysfunction (50% above baseline)
			Elevated serum lactate (2.2–4.9 mmol/l)
Peripheral white cells	Normal	10–15 × 10^9^/l	>15 × 10^9^/l

The infection control database and microbiology laboratory records from April 2008 to August 2010 were investigated to identify patients with CDI. The infection control team in the hospital maintains a prospective database. The surveillance for CDI includes the collection of patient details for each episode such as the hospital number, date of birth, and sex, as well as information concerning the patient’s location, date of admission, and care details at the time the fecal sample was taken. Every quarter the data collected in the enhanced surveillance is used to produce epidemiological data. In patients with multiple admissions to the NICU, only the first admission was used for analysis. Data on age, gender, date of admission to NICU, diagnosis, date of diagnosis of CDI, and APACHE II score were collected. The presentation, disease severity, course of disease, and complications such as colitis or time delay in discharge from ICU due to non-availability of isolation beds in receiving areas if any were noted.

The number of stool samples submitted for microbiological examination, the choice of specific antibiotic treatment for the CDAD, discontinuation or otherwise of ongoing antibiotics, and the need for more than a single course of treatment were noted. Potential risk factors associated with CDI including use of antibiotics, laxatives, anti-secretory drugs, systemic corticosteroid use, and invasive lines were recorded when the exposure was prior to the diagnosis of disease. Other causes of sepsis prior to CDAD were recorded.

Incidence rates of NICU-acquired CDAD infection were calculated as the number of cases of NICU-acquired CDAD after 48 h in intensive care divided by the total number of bed days in the study period multiplied by 10,000.

The prevalence rate was calculated as the number of NICU-acquired cases during the study period divided by the total number of NICU admissions in the study period.

All treatment for CDAD was made after discussion with a medical microbiologist including duration and discontinuation or otherwise of systemic antibiotics.

The audit was registered and approved as per institute policy which waived institute review board and patient consent in view of the nature of the study.

## Results

### Incidence and prevalence of CDAD

In the study period, nine patients acquired CDAD after 48 h of admission. A total of 2212 patients were admitted to the NICU in this period with 10,825 bed days, giving a CDAD infection incidence rate of 8.3 per 10,000 days and a prevalence of 0.4%.

### Patient characteristics

Three out of the nine patients were male (Table 2). The median age of the CDAD patients was 55 (20–72) years, with two patients>65 years of age. All of the CDAD cases were emergency admissions of which eight were neurosurgical patients and one was a neurology patient. Patients had a mean 6.7 (±5.2) days of diarrhea prior to a positive assay, with a median of 3 (1–7) stool samples having been sent for toxin assay.

**Table 2:** **Characteristics of patients with *Clostridium difficile* associated disease in the NICU**.

Characteristic	*n*	%/IQR
Age (IQR)	55	20–72
Male gender (%)	3	33
APACHE II (IQR)	20	11–32
Days in unit (IQR)	26	11–103
Days on ventilator (IQR)	20	8–96
Severity of cdad		
Mild	2	22
Moderate	4	44
Severe	3	33
Days from admission to diagnosis (IQR)	11	3–93
Treatment (*n = 9/%*)		
Nothing	0	
Metronidazole	4	
Vancomycin	2	
Both	3	
Morbidity (colitis on CT scan)	1	
Total mortality (*n* = 9)	4 (44%)	
Mortality attributable to CDAD	0	

### Identified risk factors for CDAD

Risk factors for CDAD included age>65 – 2 of the nine patients were>65 years age, antibiotics were administered to 7/9 (77%), laxatives, and proton pump inhibitors to all the patients as per the unit protocol (100%), steroids to 3/9 (33%), and indwelling medical device was required in all patients (100%). Seven of the nine patients (77%) had received mechanical ventilation prior to diagnosis for a median period of 20 (8–96) days.

One of the patients grew the highly virulent strain NAP1/BI/027 of *C. difficile* on culture – the others being (001, 018, and 014/020).

### Antibiotic usage

Seven (77%) of the patients had received some antibiotic prior to the diagnosis of CDAD. In two patients this was a single dose of surgical prophylaxis (McDonald et al., 1998). The most frequently prescribed antimicrobials prior to CDAD were cephalosporins (71%), Piperacillin/Tazobactam (28%), Vancomycin (28%), gentamicin (14%), and Meropenem (14%).

### Concurrent infection

Concurrent infections which were considered clinically significant to warrant treatment occurred in seven (77%) of the patients, three of which were ventilator associated pneumonia, four meningitis, and one septicemia. Pseudomonas species was the organism isolated most often (43%), followed by methicillin sensitive *Staphylococcus aureus* (28%), *Enterococcus* sp. (28%), and *Klebsiella* sp. (14%).

### Course of disease and treatment

At presentation, temperature>38.3 °C and WCC>15,000/cc was present in 3/9 (33% of patients. The mean serum albumin was 2.2 g/dl. All patients by definition had free stools of Bristol chart type 5 or more (stool taking the shape of the container).

Septic shock, respiratory failure, and liver failure not directly related to CDAD occurred in four, six, and two patients respectively.

Both the patients with mild and two with moderate CDAD improved with a course of oral metronidazole of 14 days. Two patients with moderate CDAD received a course of oral metronidazole followed by treatment with nasogastric vancomycin. All three patients with severe CDAD were treated with nasogastric vancomycin for 21 days two of whom relapsed and required a further course of nasogastric vancomycin.

Three patients (33%) had a perceived delay in discharge from NICU (ranging 1–8 days) due to CDAD infective status.

### Morbidity and mortality

One patient developed CDAD colitis (CT scan positive for bowel wall thickening and stranding); there were no cases of bowel perforation or GI hemorrhage directly attributable to CDAD. Of a total mortality of 4/9 cases, there was no mortality attributable to CDAD.

## Discussion

*Clostridium difficile* associated disease is an important hospital-acquired infection among critically ill patients. The basic pathogenesis involves the disturbance of the normal colonic microbial flora by antibiotics. This promotes the conversion of the *C. difficile* spore to a vegetative form and encourages the production of toxins. The usual presentation is diarrhea, which may be accompanied with abdominal pain, hypotension, electrolyte perturbations, and fever (Loo et al., 2005).

There is a wide variation in the reporting of *Clostridium* infections among the published studies. CDAD is more common with in the general intensive care setting with an overall incidence and prevalence as high as 54 per 10,000 patient days and 7% respectively (Lawrence et al., 2007; Scott et al., 2012). In the pediatric ICU, Pinto (2012) have reported a CDAD prevalence of 1.5%.

Among the limited reports of CDAD in subgroups of ICU patients, Musa et al. (2011) and Musa et al. (2010) have reported the prevalence of CDAD infection in the NICU as 0.6 and 0.5% in the cardiothoracic unit. Crabtree et al. (2011) in their study report an incidence of 7.2 per 10,000 patient days in the burns ICU (BICU) and 15.5 infections in 10,000 patient days among patients in the non-BICU in spite of longer duration of stay and greater number of surgical interventions in the BICU patients who were more often young males. The rates of infection in our patients appear to be lesser than those in general ICUs and similar to the ICUs catering to patient subgroups.

Similar to the findings of Musa et al. (2010) we had a greater proportion of emergency admissions, post-surgical patients and had a similar median age of patient population infected with similar severity of illness at admission (APACHE II scores).

The three major risk factors for CDAD are the use of antibiotics, advanced age, and prolonged hospitalization (Loo et al., 2005) (Table 3). A single-dose surgical antibacterial prophylaxis suffices to increase the risk of CDAD. Exposure to all antimicrobial classes is implicated with fluoroquinolones leading the list (Pépin et al., 2005; Carignan et al., 2008). Cephalosporin use is common in our unit for treatment and surgical prophylaxis due to their desirable CSF penetration and most of our patients 7/9 had received cephalosporin group of antibiotic at least once.

**Table 3:** **Risk factors associated with CDAD [34]**.

Perturbation of the intestinal flora/mucosa or immune system	Antibiotic treatment
	Fluoroquinolone-resistant BI/NAP1/027
	Proton pump inhibitors-receptor antagonists
	Chemotherapy (hematopoietic and H2 stem cell and solid organ transplants)
	Glucocorticoids
	Radiation treatment
	Intestinal stasis (medications)
	Abdominal surgery
	Nasogastric tubes and enemas
Environmental contamination	Length of stay in hospital or long-term care facility
	Possible: food contamination, pets, and farm animals
Host factors	Age>65 years
	Multiple comorbidities
	Peripartum women and children
	Inflammatory bowel disease
	HIV
	Chronic kidney disease requiring hemodialysis

The incidence, complications, and recurrence of CDAD among individuals aged>65 years may be up to 10 times higher than among younger adults (McDonald et al., 2006; Garey et al., 2008). Only two patients in our cohort were>65 years of age, and this may explain the lower incidence and complications. It has been argued that the prolonged length of stay in the ICU for these patients could be the reason rather than the consequence of CDAD (Wanahita et al., 2003).

The Canadian and English outbreaks of CDAD were related to the more virulent subtype-NAP1/BI/027. The one patient in our cohort infected with this strain developed CDAD colitis and required prolonged treatment with nasogastric vancomycin.

As in other such studies, a low number of patients with CDAD in our group does preclude meaningful statistical analysis, since it may show spurious associations. The findings can only be presented in a descriptive form. A larger prospectively designed study is needed wherein regression analysis can be performed to analyze the risk factors associated with patients in NICU when compared to general ICU patients. Clinical signs and symptoms of CDAD include non-specific ones of diarrhea, abdominal distention, leucocytosis, and fever. All our patients being on laxatives, and most having concurrent infections, could have influenced our categorization of disease severity and may have led to over treatment in some cases.

In conclusion, the rates of infection and morbidity in our unit are acceptably low. Among other preventive measures, early stool sampling, and isolation, repeated sampling in continued diarrhea of uncertain cause and initiating vancomycin in severe infections as followed in our unit may be of particular benefit. A high ratio of isolation beds to beds in a common space (ratio 8 side room beds:12 common beds) could be another factor helping prevent cross infection. There is a need for a larger database and greater uniformity in reporting CDAD infections in ICUs catering to special patient groups to better compare and understand the risk factors, design tailored severity scoring and lay down discharge criteria of patients with CDI from the NICU.

## Conflict of Interest Statement

The authors declare that the research was conducted in the absence of any commercial or financial relationships that could be construed as a potential conflict of interest.
